# Electronic patient-reported monitoring of symptoms during follow-up of ovarian cancer patients: a feasibility study

**DOI:** 10.1186/s12885-022-09817-5

**Published:** 2022-07-02

**Authors:** Fiona Kennedy, Leanne Shearsmith, Marie Holmes, Zoe Rogers, Rob Carter, Uschi Hofmann, Galina Velikova

**Affiliations:** 1 Section of Patient Centred Outcomes Research, Patient Reported Outcomes Group, Leeds Institute of Medical Research at St James’s, University of Leeds, Bexley Wing, Beckett Street, Leeds, LS9 7TF UK; 2 Calderdale & Huddersfield NHS Foundation Trust, Huddersfield Royal Infirmary, Acre St, Lindley, Huddersfield, HD3 3EA UK

**Keywords:** Ovarian cancer, Follow-up, Electronic patient-reported outcomes, Feasibility study, Compliance, Acceptability

## Abstract

**Background:**

Ovarian cancer patients require monitoring for relapse. Innovative follow-up methods are increasingly being explored. An electronic patient-reported outcome (ePRO) follow-up pathway was developed for women treated for ovarian cancer. This feasibility study explored patient acceptability and compliance.

**Methods:**

A single-arm non-blinded prospective feasibility study was undertaken at two hospitals. Participants were women who had completed treatment for ovarian cancer whose clinician was happy for them to be monitored remotely. Automated 3-monthly reminders were sent to participants to complete an ePRO questionnaire and obtain blood tests. Participants were reviewed over the phone by their clinical nurse specialist instead of attending clinic-based follow-up. The primary outcome was compliance (expected ePRO completions/blood tests) across the 12-month study period. Secondary outcomes were recruitment, attrition, resource use, symptom severity/alerts and patient acceptability.

**Results:**

Twenty-four women consented (50% consent rate), and 13 remained on study at 12 months. Seven women relapsed, 3 chose to withdraw, and 1 withdrew for other clinical reasons. ePRO compliance was high and consistent at 75-82%, although the two hospitals differed. Adherence to the clinical protocol was evident for blood tests and contacts with staff (fewer visits, more phonecalls compared to an earlier audit). End-of-study feedback indicated high patient satisfaction.

**Conclusions:**

Remote ePRO follow-up for ovarian cancer is feasible and acceptable to patients who are able and willing to participate. However, the low recruitment rate (ineligible + declined) indicate it is not suitable/acceptable to all patients immediately post-treatment. Further large-scale research and implementation work is required, especially in a post-COVID era.

**Trial registration:**

ClinicalTrials.gov ID: NCT02847715 (first registered 19/05/2016).

**Supplementary Information:**

The online version contains supplementary material available at 10.1186/s12885-022-09817-5.

## Introduction

The numbers of individuals living with and beyond cancer in the UK are expected to continue to rise [1], leading to more pressure on clinical services to provide safe, effective and acceptable follow-up care instead of traditional outpatient visits in secondary care [2]. New models of follow-up have been increasingly explored in research [3, 4] and clinical practice over the last decade [5, 6].

Ovarian cancer follow-up requires particular focus on detecting any potential disease relapse, which is common [7], with estimates suggesting 40-50% relapse within 12-month of first-line treatment, and ~ 25% within 6 months [8, 9]. Follow-up is traditionally done with routine face-to-face appointments for 5 years, alongside serum biomarker testing (CA125), physical examination and CT imaging where required [10]. Routine follow-up does not necessarily increase survival [11], and although reassuring for some individuals [12], can also reignite anxiety [13].

Nurse-led gynaecological follow-up has shown high patient satisfaction [4, 14–17]. Coleman & Newton [5] present a UK gynaecological clinical practice survey from 2012 to 2019 illustrating increased levels of telephone (25-32%) and patient-initiated (32-42%) follow-up (PIFU, defined as “the patient is not followed up in secondary care but seen only if the patient requests or initiates a contact”, Leeson et al. 2013, p.2). Morrison et al. [4] conducted a small UK-based randomised control trial (RCT) of a nurse-led telephone education and needs assessment-focused follow-up programme versus usual care, which demonstrated the intervention group had positive changes in quality of life (QoL) and reduced costs. It is clear that further growth in alternative follow-up is likely, especially in light of the coronavirus pandemic which has prompted calls for the rapid introduction of remote services [18]. However, it is important to evaluate the acceptability and feasibility of such methods on both patient and clinical outcomes.

Growing research evidence has shown that monitoring patient reported outcomes (PRO, defined as a patient’s own assessment of their health, [19]) is feasible and effective during and following treatment [20–23], providing benefits for communication, symptom control and QoL. Furthermore, electronic (ePRO) methods could provide additional support and rapid communication between patients and clinicians [24, 25]. An ePRO follow-up pathway enables patient’s symptoms/needs to be monitored regularly, remotely, and communicated with their clinicians, and prompt face-to-face review when symptoms/concerns are reported. ePRO follow-up services are emerging in the literature [22, 26–28]. Qualitative interview work conducted in 2018 illustrated that ovarian cancer clinicians and patients were supportive of an ePRO pathway model post-treatment, suggesting the benefits would be reduced hospital visits when well, regaining normality, but continued access to service/clinicians for reassurance when required [29]. However, further evaluation is needed to explore this follow-up in a real-world clinical context to establish feasibility and acceptability, and to inform the effective use in existing and future service provision plans.

The purpose of this feasibility study was to evaluate an ePRO follow-up pathway (‘ePRIME’, **e**lectronic **P**atient self-**R**eported outcomes to **I**mprove cancer **M**anagement and patient **E**xperiences) designed to remotely monitor and communicate patient symptoms (particularly common relapse symptoms) post-treatment in ovarian cancer. The study aimed to explore whether this pathway is feasible and acceptable alongside blood tests and telephone calls with a clinical nurse specialist (CNS). The primary outcome was compliance (expected ePRO completions and blood tests). Secondary outcomes included recruitment rates, attrition, healthcare resource use, symptom severity/alerts, and patient acceptability. 

## Methods

### Study design

A single-arm non-blinded prospective feasibility study was undertaken at two hospitals (a cancer centre and a smaller district general hospital). The study was originally planned as a multi-centre time-sequential between subjects before (audit)-after (feasibility) study in order to allow an estimation of impact of the ePRO pathway on the service use (hospital visits, blood tests) and on patient outcomes (time to detection of relapse, self-efficacy, quality of life). The original eligibility criteria included patients who had completed treatment within 6 months. However, the introduction of further maintenance treatment for ovarian cancer (i.e. niraparib) reduced the target sample for the feasibility (after) phase as patients moved onto maintenance treatment rather than routine follow-up. This change prompted the extension of the eligibility criteria to include patients who were further post-treatment, but this resulted in imbalance between the samples in the ‘before’ and ‘after’ phases, and meant the precise comparison and estimation of impact was impeded. Table 1 and the participant details section below describe the differences between the phases, and therefore this paper mainly focuses on the feasibility ‘after’ phase data.

**Table 1:** Demographic and disease characteristics between the usual care and feasibility participants

	Audit (Before) ***N =*** 45^a^	Feasibility (After) ***N =*** 24
**Hospital site**		
Hospital A	17 (37.8%)	8 (33.3%)
Hospital B	28 (62.2%)	16 (66.7%)
**Age**		
Median (range), years	62.3 (41.9-85.4)	62.8 (21.0-78.7)
**Marital Status, n (%)** ^b^		
Married / Civil Partnership	28 (62.2%)	17 (70.8%)
Co-habiting	4 (8.9%)	1 (4.2%)
Separated / Divorced	4 (8.9%)	2 (8.3%)
Widowed	2 (4.4%)	1 (4.2%)
Single	2 (4.4%)	3 (12.5%)
**Employment, n (%)**^**c**^		
Full time	9 (20.0%)	2 (8.3%)
Part time	2 (4.4%)	4 (16.7%)
Unable to work	4 (8.9%)	1 (4.2%)
Retired	23 (51.1%)	15 (62.5%)
Unemployed (not looking)	3 (6.7%)	0
Student	0	1 (4.2%)
**Education, n (%)**^**c**^		
Basic school	11 (24.4%)	6 (25.0%)
Beyond minimum school leaving age	12 (26.7%)	7 (29.2%)
Degree or professional qualification	18 (40.0%)	10 (41.7%)
**Diagnostic stage:**		
Stage 1C/2	8 (17.8%)	5 (20.8%)
Stage 3A/3B	5 (11.1%)	4 (16.6%)
Stage 3C	20 (44.4%)	14 (58.3%)
Stage 4	12 (26.7%)	1 (4.2%)
**Origin of cancer**:		
Ovary	26 (57.8%)	16 (66.7%)
Peritoneal	9 (20.0%)	4 (16.7%)
Fallopian	10 (22.2%)	4 (16.7%)
**Histology:**		
Serous	37 (82.2%)	20 (83.3%)
Clear cell	3 (6.7%)	2 (8.3%)
Endometriod	2 (3.5%)	0
Mucinous	1 (1.8%)	2 (8.3%)
Mixed	1 (1.8%)	0
Unknown	1 (1.8%)	0
**Tumour grade:**		
Poorly differentiated	39 (86.7%)	19 (79.2%)
Other (e.g. moderate, well differentiated)	4 (8.9%)	4 (16.7%)
Unknown	2 (4.4%)	1 (4.2%)
**BRCA status:**		
No abnormality	32 (71.1%)	19 (79.2%)
BRCA confirmed	7 (15.6%)	2 (8.3%)
Not tested	6 (13.3%)	3 (12.5%)
**Time since last treatment completed:**		
Median (Range), days	39.0 (4-153)	412.5 (32-3262)
< 12 months	45 (100%)	11 (45.8%)
> 12 months	0	13 (54.2%)
**Number (%) who relapsed during the study period:**	24 (53.5%)	7 (29.2%)
Median months to relapse (patient being informed) since last treatment end	6	13
Range (min-max)	10 (2-12)	44 (5-49)
**Baseline QOL outcomes, mean (s.d):**		
FACT-O total score, possible range: 0-152	118.9 (18.11)	122.2 (24.77)
FACT-G score, possible range: 0-108	85.6 (14.61)	88.3 (19.30)
- Physical subscale, possible range: 0-28	23.3 (3.79)	24.8 (3.33)
- Social subscale, possible range: 0-28	24.3 (4.26)	23.3 (7.70)
- Emotional subscale, possible range: 0-24	17.7 (4.58)	18.2 (5.31)
- Functional subscale, possible range: 0-28	20.3 (5.57)	22.0 (6.68)
EQ 5D-VAS, possible range 0-100	78.7 (13.49	84.7 (13.52)
QLACS fear of recurrence subscale, possible range 4-28	14.2 (6.20)	13.8 (5.91)
Self-efficacy total score, possible range 6-60	47.8 (10.81)	49.2 (11.68)
PAM 13 score, possible range 0-100	61.7 (13.67)	64.6 (15.68)

^a^Three audit participants did not return the baseline demographic/computer use questionnaire.

^b^Two audit participants did not answer the marital status questions.

^c^One further audit participant and one feasibility participant did not answer the employment and education questions.

*Abbreviations*: *FACT-O* Functional Assessment Cancer Therapy-Ovarian, *FACT-G* Functional Assessment Cancer Therapy-General, *EQ. 5D-VAS*, EuroQol 5 Dimension Visual Analog Scale, *QLACS* Quality of Life in Adult Cancer Survivors, *PAM* Patient Activation Measure

### Participants and setting

All feasibility study participants were recruited from outpatient oncology clinics at two hospitals from September 2018 to December 2019 (audit participants were recruited from the same clinics March 2017-September 2018). Ethical approval for the overall study was obtained from Health Research Authority (HRA) Research Ethics Committee (REC) and the individual hospitals. All study procedures, including informed written consent, were undertaken in accordance with the Declaration of Helsinki.

Eligibility criteria included age ≥ 16 years, English language fluency, provision of written informed consent (initial approach and study information were given by the clinical team), and access to the internet (computer literacy was a stipulated eligibility for the audit phase to obtain similar groups). Patients exhibiting overt psychopathology/cognitive dysfunction, participating in other clinical trials or on maintenance treatment (e.g. tamoxifen, avastin, niraparib) requiring scheduled face-to-face appointments were excluded. Patients in the audit phase had to have completed treatment within last 6 months, whereas in the feasibility phase there were no specific time requirements post-treatment completion, as long as their clinician was happy to move them onto a remote pathway.

### Procedure and description of the intervention

**Fig. 1:**
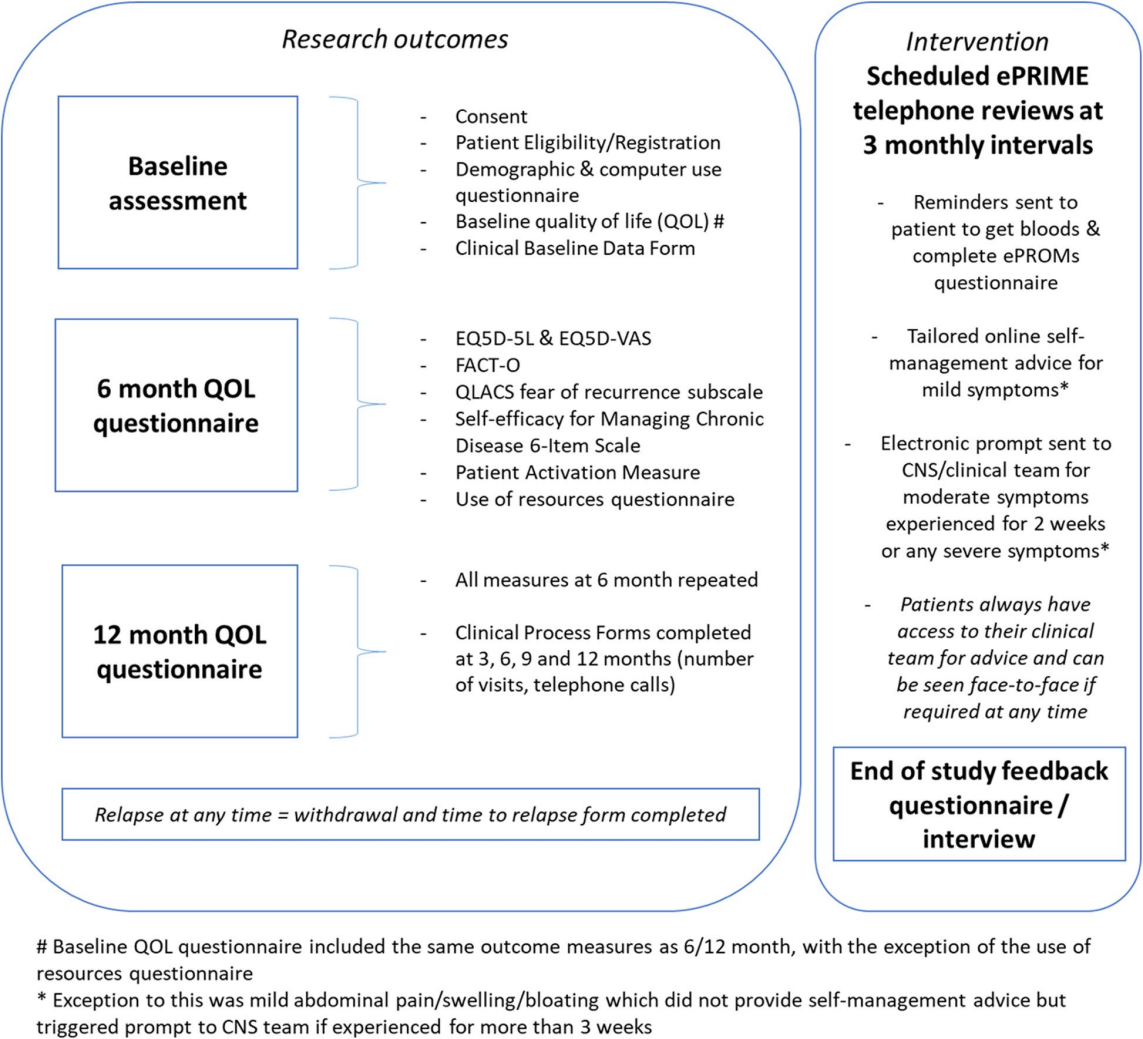
Schedule of study activities and assessments

Participants were on study for a maximum of 12-months, and study activities are outlined in Fig. 1. Baseline paper-based QoL assessments were taken at the point of recruitment (in person/via post; approx. 20-30 minutes). Repeat QoL assessments (EQ-5D-5L [30], Functional Assessment of Cancer Therapy-Ovarian [31], Fear of Recurrence subscale from the Quality of Life in Adult Cancer Survivors (QLACS) questionnaire [32], Self-efficacy for Managing Chronic Disease 6-Item Scale [33], and the Patient Activation Measure [34]) were posted to participants at 6- and 12-months. Clinical process information (number of visits, phonecalls, blood tests) was collected by researchers from medical records. Any participant who relapsed was withdrawn and no further data collected after the date of confirmed relapse. Relapse was defined as disease progression based on rising CA125 and new/progressive disease on CT, resulting in further treatment and face-to-face monitoring.

Audit participants received usual care. Feasibility phase participants received access to the intervention for a maximum of 12-months, which consisted of an ePRO web-based symptom questionnaire. Participants received a web-based demonstration of the ePRO system/questionnaire with a member of the research team (either face-to-face in clinic or via a telephone call if recruited remotely during the coronavirus pandemic), and received a paper user-guide for guidance at home, and the research team’s contact details if any issues at any time. Participants were then reminded (by email/text) to complete the ePRO questionnaire every 3 months, which was anticipated to take 15-20 minutes. As there is no ovarian cancer specific PRO for detecting relapse, the selection of eleven core symptom questions included in the ePRO was determined through a Delphi involving both clinicians and patients (see Shearsmith et al. 2020) [35]. The symptom items used were Patient Reported Adverse Event items (PRAE; 35) and examples of the items are presented in Additional file 1. Participants were asked if they had experienced each symptom during the past 2 weeks, and if they had they were asked further questions about the duration (in weeks), frequency (rarely/occasionally/frequently/almost constantly), and a free text box to add any more detail. In addition to the symptom items, participants also completed a holistic needs checklist to assess their concerns and needs [36] (e.g. emotional, confidence, work/employment, financial, travel insurance, family, psychological, relationships, or sexual). ePRO access was always available should patients wish to complete more frequently. Following clinical advice, algorithm-based email alerts (see Additional file 1) were generated to the clinical/CNS team for mild abdominal symptoms (lasting 3 or more weeks), moderate symptoms lasting for 2+ weeks (except moderate nausea, lack of appetite, fatigue, neuropathy, which were considered mild) or severe/very severe symptoms regardless of duration. Alerts were also generated if a patient wanted help with any of the holistic needs checklist issues or wished to speak to their CNS.

In addition, feasibility phase participants received a scheduled telephone call with a CNS every 3 months who had access to their ePRO data and CA125 blood test result (which women arranged at their convenience, e.g. at their general practitioner, or at hospital if preferred). Participating nurses received training (face-to-face and a reference user guide) in how to access the electronic ePRO information (Additional file 2). The ePRO intervention was instead of receiving standard face-to-face outpatient appointments.

Following study completion, attempts were made to invite all participants to provide feedback on the ePRO intervention via an end-of-study feedback questionnaire and/or in-depth interview (the latter is described elsewhere).

### Analysis of outcomes

A formal sample size calculation was not undertaken but the aim was for a sample large enough to detect small or medium effects suggested by Whitehead et al. [37] who indicate a sample of *n =* 25 or *n =* 15 respectively is required. Descriptive statistics were used to describe the demographic and clinical variables, study recruitment/compliance data and responses to acceptability feedback questions.

Compliance to the intervention (number of ePRO completions/number of expected ePRO completions, number of blood tests/expected blood tests) was the primary outcome. Following any withdrawal (patient choice, relapse or other reason), the date of withdrawal determined the point after which completions were not expected. Participants were also categorised into those who ‘completed’ the 12-month study versus ‘left study’. All online completions were categorised into expected (proximal to date of scheduled CNS review) or additional/unscheduled (not near review). If there was no completion near the CNS review, this timepoint was marked as missed.

Secondary outcomes included patient recruitment (consent rate, number of eligible who declined), retention/withdrawals (number, timing, reason), severity of ePRO symptoms and number of alerts. Clinical process data in terms of healthcare resource use was summarised by the total number of visits, phonecalls, all hospital contacts (visits + phonecalls). We also calculated the number of weeks on study per visit/phonecall/contact (e.g. weeks on study divided by total number of visits) in order to factor in the time on study. Note, one participant was excluded from the clinical process data analysis as they withdrew on day 5. Patient acceptability was assessed by exploring the end-of-study questionnaire data.

## Results

### Participant characteristics

Twenty-four participants were recruited to the feasibility phase (*n =* 8 from hospital A and *n =* 16 from hospital B) and 45 to the audit phase (*n =* 17 hospital A, *n =* 28 hospital B) (Table 1).

The median age of feasibility participants was 62.8 years (range 21.0-78.7). The audit and feasibility participants were similar on most demographic characteristics. However, as expected, median time from treatment completion to recruitment was 412 days for the feasibility study, compared to 39 days in the audit group. Diagnostic stage also differed with 26.7% (*n =* 12) of audit participants having stage 4 disease, compared to 1 (4.2%) feasibility participant.

All participants were also surveyed for their computer use experiences (Additional file 3), which illustrates relatively experienced computer users, particularly amongst feasibility participants (*n =* 22, 91.7% used computer for 5+ years; *n =* 19, 79.2% on daily basis), although some reported having general difficulties (*n =* 6 sometimes difficult/*n =* 1 difficult).

### Recruitment

**Fig. 2:**
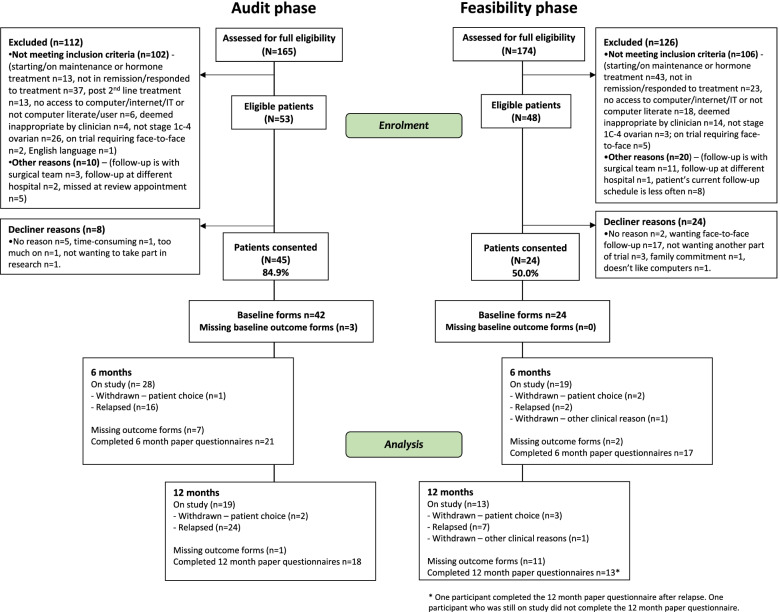
Consort diagram

Almost three-quarters of the screened potential feasibility patients were ineligible (126/174 = 72.4%, see Fig. 2) due to starting maintenance treatment such as bevacizumab/niraparib (*n =* 40) or hormone therapy (*n =* 3), having not responded to treatment (*n =* 23), no access to computer/internet/IT or not being computer literate (*n =* 18; split further as *n =* 8 not having or using computer/IT, *n =* 3 doesn’t use internet, *n =* 3 not computer literate, and *n =* 2 no further detail recorded), deemed inappropriate by clinician (*n =* 14) or being followed up elsewhere (*n =* 12).

Only 50% of eligible feasibility patients consented (*n =* 24/48). Reasons for declining centred on wanting face-to-face follow-up (*n =* 17/48, 35.4%). Other reasons for declining included preferring annual reviews (*n =* 2/24), not wanting community/general practitioner bloods (*n =* 1/24), family commitment (*n =* 1/24), disliking computers (*n =* 1/24) or no reason provided (*n =* 2/24). The audit phase had a higher consent rate (*n =* 45/53, 84.9%).

### Compliance to the intervention

#### Compliance with ePRO completions

**Fig. 3:**
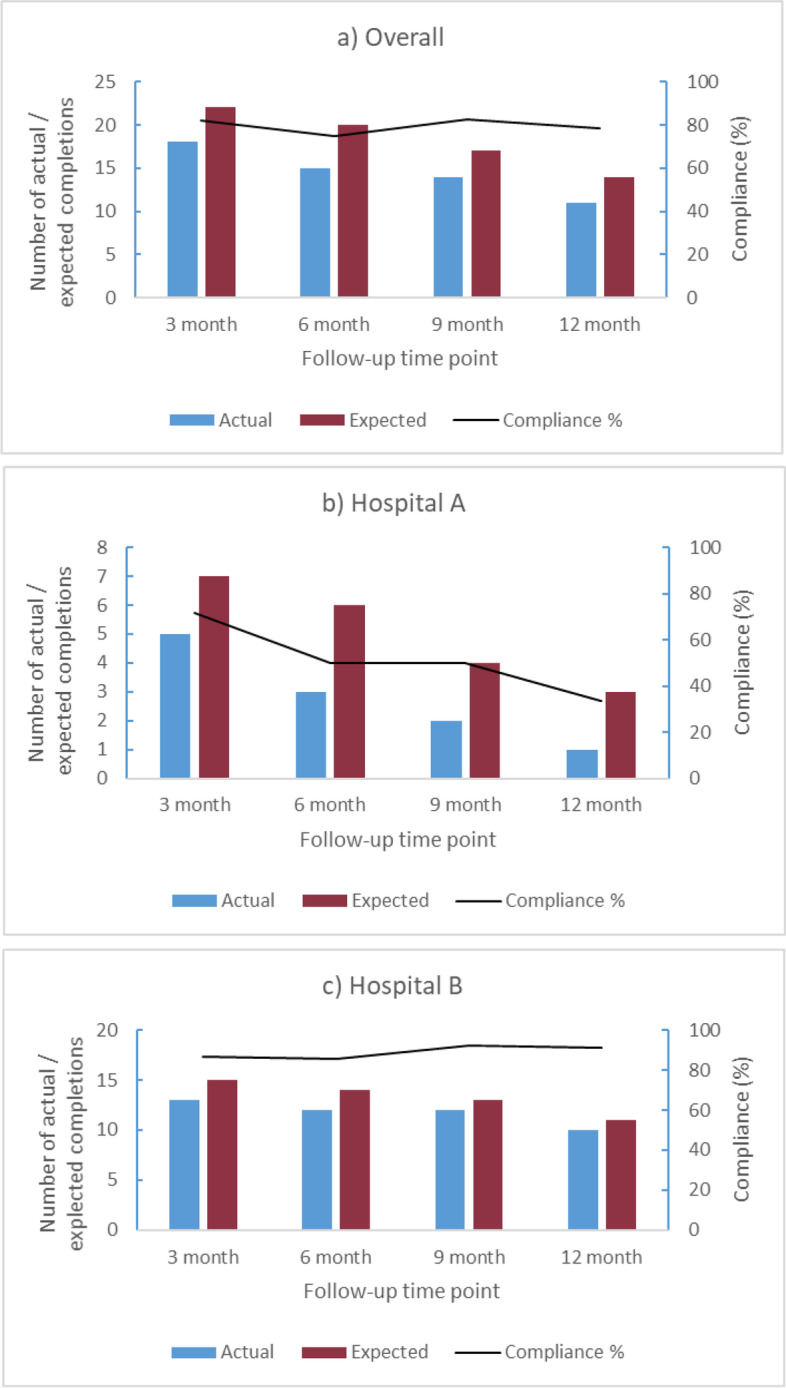
Actual, expected and percentage compliance at each time point overall (**a**), and for each hospital site (**b**, **c**)

Overall, there were 78 ePRO completions during the feasibility study, and the total number per participant ranged from 0 to 9. The scheduled/unscheduled categorisation illustrates (Additional file 4) that three-quarters of completions (74.4%) were scheduled, and 25.6% unscheduled (hospital A 64.7% vs 35.3%; hospital B 77.0% vs 23.0%). Fig. 3 presents the ePRO compliance (number completed/expected) in each scheduled review period (3-monthly) overall, and by hospital site, which is high and consistent at between 75 and 82%, although different patterns are evident between the hospitals (full data in Additional file 5).

**Fig. 4:**
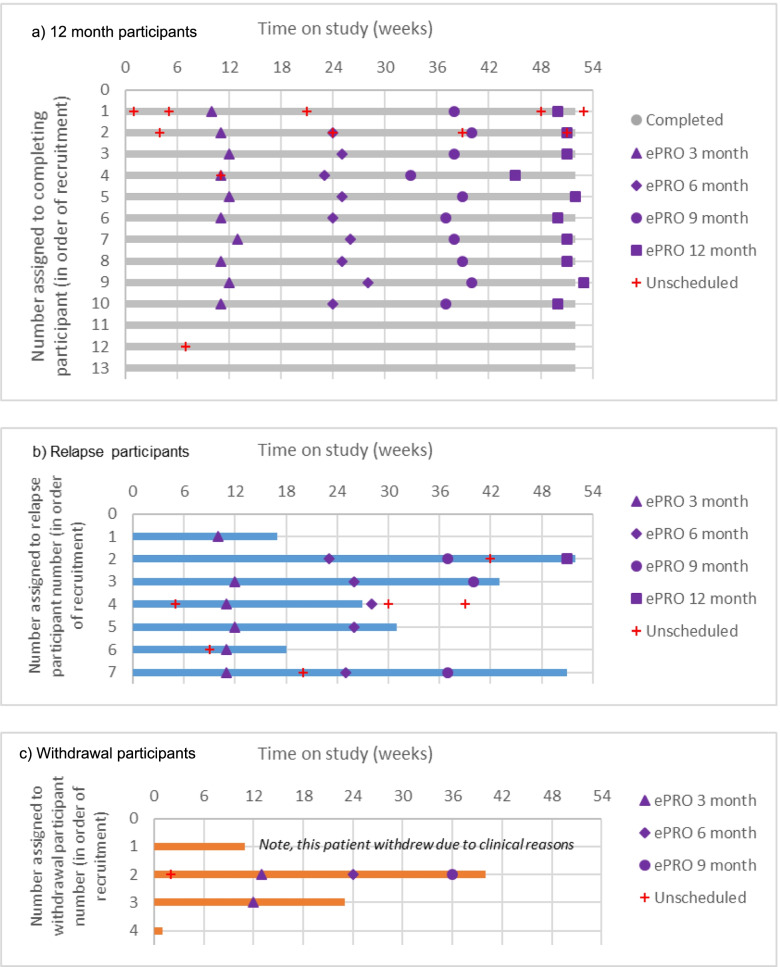
Swimmer plot illustrating all scheduled (3, 6, 9, 12 month, purple symbols) and any unscheduled completions (red cross) across study period for each participant presented by the three study status categories: (**a**) completed 12 month study, (**b**) relapsed, (**c**) withdrawn. **a** 12 month participants. **b** Relapse participants*.* **c** Withdrawal participants

To explore further the patterns of completions per participant, Fig. 4 presents a swimmers plot showing the timing of completions (scheduled and unscheduled) for each participant by their study status (completed, relapsed, withdrew). This illustrates that most participants completed regularly, apart from the last 3 participants who were non-compliant (note, all 3 were predominantly on study during the coronavirus pandemic).

### Compliance with blood tests

Fifteen participants (65.2%) had 4 or more blood tests during the study, indicating adherence to the clinical protocol (3-monthly tests). Withdrawals and change in blood test protocol during the COVID-19 pandemic affected some participants’ total number of tests. The number of tests were also influenced by study status; those who left the study had more frequent tests every 8.41 weeks versus 13.80 weeks. This pattern is expected as 70% of those who left had relapsed, and extra blood tests would be undertaken to investigate prior to relapse confirmation.

### Withdrawals and relapses

Three feasibility participants (3/24, 12.5%) requested withdrawal, all from hospital A. The reasons (and timing) included finding the QoL questionnaire stressful (withdrawal on day 5), wanting face-to-face (day 161) and not receiving the email reminders which caused confusion/disliking the remote aspect (day 285). Furthermore, another single patient was withdrawn from hospital B on clinical advice (day 77).

Table 1 includes the relapse data for both groups. Seven feasibility participants had a confirmed relapse (29.2%) with similar proportionate rates at both hospitals (hospital A = 2/8 = 25%; hospital B = 5/16 = 31.3%), with a median of 13 months (range 5-49) between last treatment end and relapse (date of patient being informed). A higher proportion of audit phase participants (*n =* 24, 53.5%) relapsed on study, with lower median of 6 months (range 2-12).

### Resource use

On average feasibility participants had 1 face-to-face hospital visit during the study (range 0-4). Six women who completed the 12-month study had no visits, ten had one visit (3 of whom relapsed), four had two visits (2 relapsed), two had three visits (1 relapsed) and one who relapsed had four visits. The number of phonecalls varied between 0 and 10, mean 5.57 and median 6. Most phonecalls were CNS-instigated (*n =* 90, 70.3%), which is expected with the scheduled 3-monthly CNS intervention call. Thirty (23.4%) phonecalls were patient-instigated, and only seven were doctor-instigated amongst three participants (of note, two of these participants received three doctor-instigated phonecalls, which appears to be the routine telephone follow-up provision for all patients during the coronavirus pandemic, rather than the intervention).

Table 2 shows the numbers and rates of hospital visits, phonecalls and overall hospital contacts during each study phase. Feasibility participants had less frequent visits and slightly more phonecalls, but the overall number/frequency of all hospital contacts was similar (Audit every 7.6 weeks vs Feasibility every 7.8 weeks). Furthermore, there were differences between the hospitals, likely reflecting different existing pathways and resources: hospital B feasibility participants had less frequent visits, more frequent phonecalls and higher frequency of overall contact with the clinical service compared to hospital A.

**Table 2:** Clinical process data by study phase - feasibility and audit participants

	All participants		Completed 12-month study		Left study		Hospital A participants		Hospital B participants	
	Audit ***N =*** 44^**a**^	Feasibility ***N =*** 23 ^**b**^	Audit ***N =*** 19	Feasibility ***N =*** 13	Audit ***N =*** 25^**b**^	Feasibility ***N =*** 10^**b**^	Audit ***N =*** 16^**a**^	Feasibility ***N =*** 7^**b**^	Audit ***N =*** 28	Feasibility ***N =*** 16
Total number (mean) of visits	163 (3.7)	28 (1.2)	76 (4.0)	10 (0.8)	87 (3.5)	18 (1.8)	55 (3.4)	10 (1.4)	108 (3.9)	18 (1.1)
*Mean number of weeks per visit*	*9.9*	*22.5*	*13.8*	*23.3*	*7.0*	*21.3*	*11.7*	*12.3*	*8.9*	*26.9*
Total number (mean) of phonecalls	75 (1.7)	128 (5.6)	32 (1.7)	78 (6.0)	43 (1.7)	50 (5.0)	11 (0.7)	24 (3.4)	64 (2.3)	104 (6.5)
*Mean number of weeks per phonecall*	*10.6*	*9.1*	*13.9*	*9.9*	*8.1*	*8.0*	*10.7*	*11.5*	*10.5*	*8.0*
Total number (mean) of contacts (visits + phonecalls)	238 (5.4)	156 (6.8)	108 (5.7)	88 (6.8)	130 (5.2)	68 (6.8)	66 (4.1)	34 (4.9)	172 (6.1)	122 (7.6)
*Mean number of weeks per contact*	*7.6*	*7.8*	*10.8*	*9.0*	*5.2*	*6.2*	*10.2*	*9.4*	*6.1*	*7.1*

^a^ Excluded 1 participant who withdrew on day 16

^b^ Excluded 1 participant who withdrew on day 4

### ePRO data and alerts

Across the total 78 ePRO symptom reports completed (with a total of 1092 symptoms reported), the presence of constipation (36/78 = 46.8%) and fatigue (36/78 = 46.8%) were the most frequent symptoms (Additional file 6), whereas diarrhoea, urinary burning and vomiting were infrequent. Overall, there were no very severe symptoms (Additional file 7), and only eight severe symptoms (*N =* 8/1092, 0.7%), namely a single severe response for abdominal pain, abdominal swelling/bloating, constipation, appetite loss, shortness of breath, neuropathy, and two responses for severe urinary urgency. Most reported symptoms were not present (*N =* 822/1092, 75.3%), mild (*N =* 186/1092, 17.0%) or moderate (*N =* 66/1092, 6.0%).

Amongst the seven participants who relapsed, three patterns of relapse detection emerged. Three patients had rising CA125 with no symptoms when CT was requested, two had rising CA125 plus symptoms, and for two the reported symptoms alone prompted the CT (one had slightly raised CA125). Amongst the four participants who experienced symptoms, one patient completed their last ePRO 11 weeks earlier (no symptoms) but attended A&E with acute symptoms. The other three patients recorded symptoms on their last ePRO prior to relapse confirmation. Two of these patients also submitted additional/unscheduled ePRO reports (see case study in Additional file 8), illustrating that they fully engaged with using the system as a way of communicating symptoms and concerns with their clinical team.

In 38/76 (50%) full completions (of note 2 completions were partial and did not complete the holistic needs section) at least one holistic need was reported, and overall 76 individual holistic needs were ticked by participants (Additional file 9). The most frequent holistic need was emotional needs (e.g. worrying and anxiety; *n =* 30/76, 39.5%), and a small number having psychological (e.g. depression) needs (*n =* 10/76, 13.2%). Other holistic needs were reported less often (between 3 and 6 times). Only 8 of the 38 (21.1%) participants reporting holistic needs indicated wanting support for these needs.

Alerts were generated from 49/78 (62.8%) ePRO completions, with a total of 105 individual item alerts (alerts were generated at an individual question level). The content of individual alerts is detailed in Additional file 10. Around a quarter (25 alerts) were not symptom-related alerts, but requests to speak to the CNS (*n =* 17) or wanting holistic support (*n =* 8). The most common symptom-related alerts were abdominal swelling/bloating (*n =* 15), abdominal pain (*n =* 14), ‘other’ symptoms (free text response, *n =* 13) and constipation (*n =* 9).

### Acceptability

QoL outcome data (baseline, 6 and 12 months) was successfully collected and showed similar scores at baseline across the feasibility and audit participants (Table 1) and stable scores overtime (Additional file 11).

Eighteen participants provided end-of-study feedback (75.0%; 12 who completed 12 months, five who relapsed, and one who chose to withdraw), whereas six participants did not provide feedback. Two of the non-completers had relapsed, and three had withdrawn (two had only been on study < 3 months and not used the system). Full feedback data is provided in Additional file 12. Overall most participants found the system very easy or easy to learn to use, to access and answer the symptom questions. Three participants found ‘very few questions relevant’ and three found it difficult/very difficult to arrange a blood test (3/18 = 16.7%). The time it took to complete the ePRO was considered ‘about right’ by 17 (94.4%, the remaining participant left this question blank). The mean actual time for completion was 12.35 minutes (median 11, ranging from 1:20 to 31:37 minutes). Most participants were very happy/relatively happy (72.2%/16.7%) to be monitored this way (*n =* 2, 11.1% were neither happy nor unhappy). In terms of future use, 15/18 (83.3%) would be happy to be remotely followed again (*n =* 1 would not, *n =* 2 unsure), and 14/18 (77.8%) would recommend to other patients (*n =* 4 unsure, two in each hospital).

## Discussion

This study represents one of the first attempts at using and evaluating an ePRO system during follow-up after ovarian cancer treatment (10) and was specifically designed to focus on monitoring common relapse symptoms and tumour marker levels, and support women during follow-up. It is important to note that ovarian cancer is mainly a disease of older women, and therefore evaluating the feasibility of a web-based electronic system in this context is very important [38]. The results demonstrate a mixed picture, with high ineligibility in ovarian patients immediately post-treatment, 50% consent rate, but low attrition (3/24 = 12.5%), high compliance (~ 75-82% of expected ePRO completions) and positive feedback amongst participants.

The lower consent rate than routinely observed rates in PRO studies typically 70%, [21], is not unsurprising given that the study required patients to agree to a significant change to their follow-up (no routinely scheduled face-to-face visits) and is similar to Morrison et al’s (2018) RCT of alternative nurse-led follow-up [4]. Clearly, for some patients this shift, despite the carefully planned system to monitor symptoms, regular phonecalls and continued access to the clinical team, was not acceptable. Further work should explore whether there is a pinnacle time post-treatment where acceptance is higher or if additional support elements would make it more appealing (e.g. video-based consultations, or initial utilisation of the system whilst on treatment). However, the high rate of ineligible patients may indicate that ovarian cancer is not the most suitable group for this type of follow-up, with low rates of clinical suitability owing to the recent widespread introduction of long-term maintenance treatments, and some patients not having access to computer/internet or lacking the confidence to use IT-based systems. However, half of eligible patients did find it acceptable, and this method may be suitable if future patients can be monitored remotely whilst on maintenance treatments. Clearly clinical practice has since been forced to change dramatically during the coronavirus pandemic, but the importance of careful risk stratification of any new follow-up methods remains important [39]. However, patient and staff attitudes and willingness to engage with remote/web/telephone-based follow-up methods may have changed during the pandemic [40, 41], and recruitment rates may differ in a post-COVID era. The current study completed recruitment before the pandemic, but 10 women were actively on study in March 2020, and commented on benefits of ePRO whilst they were shielding during the first UK lockdown.

Compliance was consistently very high throughout (75-82%), but higher at one hospital. Additionally, the three patients who withdrew were from the hospital with lower compliance. There are likely to be a multitude of reasons behind these observed differences, including staffing ratios/workload, staff engagement, hospital/research nurse resources, and IT issues that affected the reminders and access to results by clinicians at that hospital. In particular, the core study researchers were based at the hospital that had higher compliance, and the ease of support and access/communication between the study researchers and CNS team was evident. This illustrates the importance of in-depth consideration of all the resources required (e.g. clinician engagement, administrative support and training) if this type of follow-up is taken forward into routine clinical practice [42].

The high compliance and the positive feedback amongst the majority of women illustrates an overall acceptance about using remote follow-up methods in this setting for at least some patients. Most participants found the eleven core symptom questions easy/very easy to answer, highlighting acceptance of the PRAE items [23, 43] amongst women previously treated for ovarian cancer. Furthermore, using these items within the system algorithm appeared to work well, and further work should validate their use in this context. Areas of improvement indicated by the feedback questionnaires included accessibility of general practitioner bloods, which illustrates further work is required to facilitate integration and communication of follow-up between primary and secondary care [44]. Furthermore, the high rate of completions that generated alerts to the clinicians (62.8%, see Additional file 10 for detail of symptom and non-symptom alerts), may suggest that refinement of the criteria is required to make this feasible for clinical practice. However, views of the volume of alerts first needs to be explored with the clinicians who received these alerts. In-depth qualitative interviews conducted with 16 women and the 4 CNS are presented separately to comprehensively explore the experiences of both groups (Kennedy et al. unpublished manuscript). This interview work, and the low recruitment rate (high decliners) presented in this paper, highlight that the timing of approach is important and a single approach may not suit all patients. Further implementation work is required.

The limited comparison with the audit phase data illustrate that the intervention successfully reduced the number of in-person hospital visits, whilst increasing telephone contact. The large discrepancy between relapse rates in the two phases (53.5% audit, 29.2% feasibility) may reflect that many relapses occur early, within 18 months [45], or that clinicians were more careful and selective of approaching women for the feasibility phase. Further work would be required to specifically explore the effectiveness and estimate the impact of this method of follow-up on relapse outcomes.

A major limitation to this study is the lack of a comparable control group. We originally opted for a before-after design to accommodate the time required to develop the intervention [35, 29] within the time-limited funding, aiming to allow us to collect usual care data alongside the development work. However, this meant we faced a historic effect [46], with the difficulties of changing maintenance treatment policy over time, resulting in the need to change eligibility criteria and incomparable groups. This suggests that a RCT would be advisable for future exploration of this area. Further limitations of this work include that, despite efforts to obtain, there was a lack of feedback from those who chose to withdraw, making it difficult to comprehensively conclude the views of those who tried but did not find the intervention acceptable. The findings may also be affected by racial and/or economic disparities as participants had to have access to a computer and be able to answer English language questionnaires.

This work highlights various implementation considerations for future use of remote follow-up pathways, including the need to carefully consider further the timing of introduction to patients, the importance of support from and communication with primary care, and adequate administrative and IT support.

## Conclusions

There is growing evidence of the benefits of online symptom monitoring during treatment (22, 23). A recently published RCT [23] that included ovarian patients being treated with chemotherapy suggested improvements in self-efficacy and physical well-being in the online symptom reporting group. There is less evidence of the benefits during follow-up and as an alternative to face-to-face visits, but with patients living longer post-treatment, there is a need for pilot studies to explore the feasibility of remote monitoring follow-up methods in clinical practice. This small-scale feasibility study demonstrated high compliance, generally strong patient acceptance and positive feedback of a remote follow-up pathway after treatment for ovarian cancer. However, some feedback indicated this type of follow-up may work better for patients who have adjusted somewhat post-treatment. Furthermore, if extended to patients on maintenance treatments, this would require the symptom items to be refined to capture toxicity as well as disease progression. Flexible approaches for remote symptom monitoring during follow-up are likely to be needed in the future, and therefore further careful developments and robust evaluations are warranted.

## Supplementary Information

**Additional file 1: Additional file 1a**. Algorithm options programmed on the system. The specific symptom items are presented on the left side of the figure and are also presented in the Table below. **Table 1b.** Examples of the Patient Reported Adverse Events Measure (PRAE) items used and other content of ePRO questionnaire

**Additional file 2.** The system/s architecture and access to the ePRO data by the CNS staff

**Additional file3.** Computer use/experience of participants between the usual care and feasibility participants

**Additional file 4.** Percentage of scheduled versus unscheduled ePRO completions

**Additional file 5.** Compliance with ePRO completions (proportion of completed/expected)

**Additional file 6.** Severity of symptoms reported amongst the feasibility study online responses

**Additional file 7.** Overall collective severity levels across all completions

**Additional file 8. **Case study example of a patient’s use of system and presentation in electronic patient record for clinicians to review. **8a** Tabular format, contains free text on additional symptoms or detail that patient wishes to add (most recent completion is on the left). **8b** Graphic format to track symptoms over time (most recent completion is on the right)

**Additional file 9.** Overall reported holistic needs across all ePRO completions (76* full reports)

**Additional file 10.** Individual alerts based on topic and number of alerts recorded (overall, by hospital site)

**Additional file 11.** Quality of life outcome data for feasibility phase participants

**Additional file 12. **End-of study (EOS) feedback questionnaires (completed by *n*=18 feasibility participants): a visual summary and full data table

## Data Availability

The anonymised data collected during the current study are stored at the Patient Centred Outcomes Research group. These datasets are not publicly available, but the corresponding author may consider reasonable requests.

## Abbreviations

CNS: Clinical nurse specialist
ePRO: Electronic patient reported outcomes
QoL: Quality of life
RCT: Randomised control trial
