# Supplementary material for: Electronic patient-reported monitoring of symptoms during follow-up of ovarian cancer patients: a feasibility study
Source: BMC Cancer. 2022 Jul 2;22:726. doi: 10.1186/s12885-022-09817-5 (PMC9250717; doi:10.1186/s12885-022-09817-5)
Supplement: Supplementary file 1 [file 12885_2022_9817_MOESM10_ESM.docx]

Additional file 10: Individual alerts based on topic and number of alerts recorded (overall, by hospital site)

	**Overall**	**Hospital A**	**Hospital B**
Request to speak to CNS	17	3	14
Abdominal swelling / bloating	15	2	13
Abdominal pain	14	4	10
Other symptoms (free text response, which patient rated as severe/very severe). *Most common being: tinnitus (n=4), backache (n=2), cramp (n=2).*	13	4	9
Constipation	9	6	3
Weight change	9	2	7
Additional holistic needs	8	0	8
Urinary urgency	8	2	6
Shortness of breath	6	0	6
Urinary frequency	3	1	2
Vomiting	2	1	1
Swollen leg	1	0	1
**Total**	105	25	80
