# Supplementary material for: Electronic patient-reported monitoring of symptoms during follow-up of ovarian cancer patients: a feasibility study
Source: BMC Cancer. 2022 Jul 2;22:726. doi: 10.1186/s12885-022-09817-5 (PMC9250717; doi:10.1186/s12885-022-09817-5)
Supplement: Supplementary file 2 [file 12885_2022_9817_MOESM11_ESM.docx]

Additional file 11: Quality of life outcome data for feasibility phase participants

	Baseline<br>N=24	6 months<br>N=17	12 months<br>N=13
**% Completed as expected QOL outcome questionnaires (completed/expected):** [#]	100.0%<br>(24/24)	89.5%<br>(17/19)	92.3%<br>(12/13)
**QOL outcome scores:**	**Mean (s.d)**	**Mean (s.d)**	**Mean (s.d)**
FACT-O total score<br>*Score range:* 0-152	122.17 (24.77)	111.40 (34.03)[*]	113.09 (30.41)[$]
FACT-O trial outcome index<br>*Score range:* 0-100	80.66 (15.04)	72.46 (24.69)[*]	77.02 (19.39)
EQ5D-VAS - mean (s.d)<br>*Score range 0-100*	84.71 (13.52)	79.06 (19.77)[#]	83.31 (16.21)
QLACS fear of cancer recurrence - mean (s.d)<br>*Score range 4-28*	13.79 (5.91)	13.53 (6.59)	15.46 (6.37)
Self-efficacy total<br>*Score range 6-60*	49.17 (11.68)	43.71 (17.93)	48.15 (14.83)
PAM 13 score<br>*Score range 0 -100*	64.61 (15.68)	64.59 (19.48)	61.46 (14.28)
FACT-G score<br>*Score range:* 0-108	88.29 (19.30)	78.45 (25.83)[*]	79.64 (23.93)[$]
Ovarian subscale total score<br>*Score range:* 0-44	33.88 (6.43)	33.25 (8.71)	32.42 (7.74)
Physical subscale<br>*Score range:* 0-28	24.76 (3.33)	21.25 (7.72)[*]	24.00 (4.88)
Social subscale<br>*Score range:* 0-28	23.30 (7.70)	21.54 (7.49)	20.57 (7.73)[$]
Emotional subscale<br>*Score range:* 0-24	18.21 (5.31)	17.75 (5.05)[*]	15.69 (5.25)
Functional subscale<br>*Score range:* 0-28	22.03 (6.68)	18.41 (8.47)	20.60 (9.19)

[#] This excludes any withdrawals/relapses.

[*] Due to missing answers for these questions n=16 rather than n=17

[$] Due to missing answers for these questions n=12 rather than n=13
