# Supplementary material for: Electronic patient-reported monitoring of symptoms during follow-up of ovarian cancer patients: a feasibility study
Source: BMC Cancer. 2022 Jul 2;22:726. doi: 10.1186/s12885-022-09817-5 (PMC9250717; doi:10.1186/s12885-022-09817-5)
Supplement: Supplementary file 3 [file 12885_2022_9817_MOESM12_ESM.docx]

Additional file 12: End-of study (EOS) feedback questionnaires (completed by n=18 feasibility participants): a visual summary and full data table

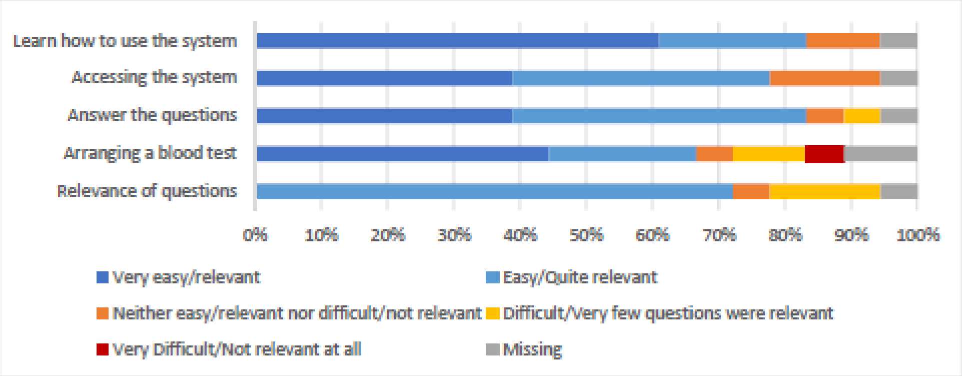

Full EOS feedback results (n=18)

	Overall N=18		Hospital A N=5		Hospital B N=13	
**1. How easy or difficult was it to learn how to use the system?**	**n**	**%**	**n**	**%**	**n**	**%**
Very easy	11	61.1%	4	80.0%	7	53.8%
Easy	4	22.2%	1	20.0%	3	23.1%
Neither easy nor difficult	2	11.1%	0	0	2	15.4%
Difficult	0	0	0	0	0	0
Very Difficult	0	0	0	0	0	0
Missing	1	5.6%	0	0	1	7.7%
**2. How easy or difficult did you find accessing the system? E.g. finding the website and logging in**						
Very easy	7	38.9%	2	40.0%	5	38.5%
Easy	7	38.9%	2	40.0%	5	38.5%
Neither easy nor difficult	3	16.7%	1	20.0%	2	15.4%
Difficult	0	0	0	0	0	0
Very Difficult	0	0	0	0	0	0
Missing	1	5.6%	0	0	1	7.7%
**3. How easy or difficult was it to answer the questions?**						
Very easy	7	38.9%	2	40.0%	5	38.5%
Easy	8	44.4%	2	40.0%	6	46.2%
Neither easy nor difficult	1	5.6%	1	20.0%	1	7.7%
Difficult	1	5.6%	0	0	0	0
Very Difficult	0	0	0	0	0	0
Missing	1	5.6%	0	0	1	7.7%
**4. How did you feel about the amount of time it took to complete the questionnaires?**						
Too long	0	0	0	0	0	0
About right	17	94.4%	5	100.0%	12	92.3%
Too quick	0	0	0	0	0	0
Missing	1	5.6%	0	0	1	7.7%

**5. How relevant were the questions to you?**						
Not relevant at all	0	0	0	0	0	0
Very few questions were relevant	3	16.7%	0	0	3	23.1%
Neither relevant nor irrelevant	1	5.6%	0	0	1	7.7%
Quite relevant	13	72.2%	5	100.0%	8	61.5%
Very relevant	0	0	0	0	0	0
Missing	1	5.6%	0	0	1	7.7%
**6. What did you think about completing the online ePRIME symptom questionnaire every 3 months?**						
Definitely too often	0	0	0	0	0	0
A little bit too often	0	0	0	0	0	0
Unsure	3	16.7%	1	20.0%	2	15.4%
I was happy to complete them every 3 months	9	50.0%	3	60.0%	6	46.2%
I would have been happy to complete them more often	4	22.2%	1	20.0%	3	23.1%
Missing	2	11.1%	0	0	2	15.4%
**7. Did you find the general advice given at the end of the questionnaire useful?**						
Very useful	7	38.9%	3	60.0%	4	30.8%
A little useful	5	27.8%	2	40.0%	3	23.1%
Unsure	4	22.2%	0	0	4	30.8%
Not very useful	0	0	0	0	0	0
Not useful at all	0	0	0	0	0	0
Missing	2	11.1%	0	0	2	15.4%
**8. Did you use any of the links provided within the advice on the system? (e.g. Macmillan or Cancer Research UK websites)**						
Yes	2	11.1%	1	20.0%	1	7.7%
No	14	77.8%	3	60.0%	11	84.6%
Not sure	1	5.6%	1	20.0%	0	0
Missing	1	5.6%	0	0	1	7.7%
**9. Were there any times when you missed completing the online symptom questionnaire?**						
Yes	4	22.2%	2	40.0%	2	15.4%
No	11	61.1%	3	60.0%	8	61.5%
Missing	3	16.7%	0	0	3	23.1%
**10. How easy or difficult was it to arrange a blood test locally? (GP surgery or local hospital)?**						
Very easy	8	44.4%	4	80.0%	4	30.8%
Easy	4	22.2%	1	20.0%	3	23.1%
Neither easy nor difficult	1	5.6%	0	0	1	7.7%
Difficult	2	11.1%	0	0	2	15.4%
Very Difficult	1	5.6%	0	0	1	7.7%
Missing	2	11.1%	0	0	2	15.4%
**11. How happy were you with being followed up and monitored in this way rather than attending a clinic appointment in person?**						
Very happy	13	72.2%	3	60.0%	10	76.9%
Relatively happy	3	16.7%	1	20.0%	2	15.4%
Neither happy nor unhappy	2	11.1%	1	20.0%	1	7.7%
Unhappy	0	0	0	0	0	0
Very unhappy	0	0	0	0	0	0
Missing	0	0	0	0	0	0
**12. Would you be happy to be followed up in this way again, now or in the future?**						

Yes	15	83.3%	4	80.0%	11	84.6%
No	1	5.6%	0	0	1	7.7%
Not sure	2	11.1%	1	20.0%	1	7.7%
Missing	0	0	0	0	0	0
**13. Did you have any direct contact with your clinical team relating to your ePRIME symptom information or blood test results?**						
Yes	14	77.8%	4	80.0%	10	76.9%
No	4	22.2%	1	20.0%	3	23.1%
Missing	0	0	0	0	0	0
**14. To what extent do you feel that the symptom questionnaire information was useful for the doctors and nurses you had contact with following your treatment?**						
Very useful	10	55.6%	2	40.0%	8	61.5%
A little useful	1	5.6%	1	20.0%	0	0
Unsure	7	38.9%	2	40.0%	5	38.5%
Not very useful	0	0	0	0	0	0
Not at all useful	0	0	0	0	0	0
Missing	0	0	0	0	0	0
**15. Would you recommend the ePRIME system to other patients?**						
Yes	14	77.8%	3	60.0%	11	84.6%
No	0	0	0	0	0	0
Not sure	4	22.2%	2	40.0%	2	15.4%
Missing	0	0	0	0	0	0
