# Supplementary material for: Electronic patient-reported monitoring of symptoms during follow-up of ovarian cancer patients: a feasibility study
Source: BMC Cancer. 2022 Jul 2;22:726. doi: 10.1186/s12885-022-09817-5 (PMC9250717; doi:10.1186/s12885-022-09817-5)
Supplement: Supplementary file 4 [file 12885_2022_9817_MOESM1_ESM.docx]

Additional file 1a: Algorithm options programmed on the system. The specific symptom items are presented on the left side of the figure and are also presented in the Table below.

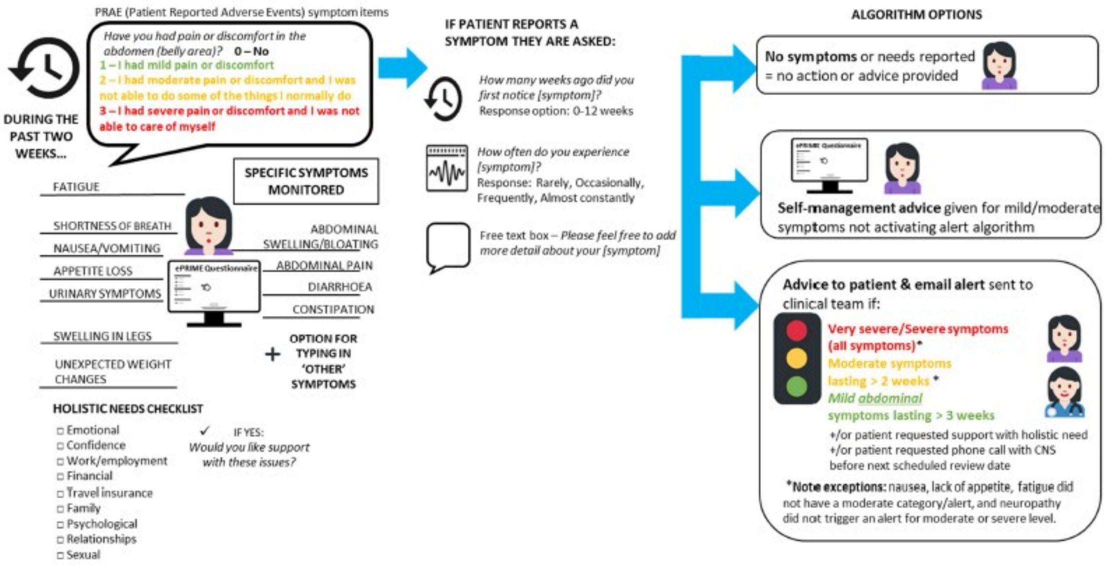


**Table 1b. Examples of the Patient Reported Adverse Events Measure (PRAE) items used and other content of ePRO questionnaire**

| (All questions are preceded with "During the past two weeks:") | | |
|---|---|---|
| **Abdominal pain** | Have you had pain or discomfort in the abdomen (belly area)? | No |
| | | I had mild pain or discomfort |
| | | I had moderate pain or discomfort and I was not able to do some of the things I normally do (for example, household chores/shopping) |
| | | I had severe pain or discomfort and I was not able to care for myself (getting out of bed, bathing, dressing) |
| **Bloating/Swelling** | Have you had swelling/bloating (uncomfortable fullness) of the abdomen? | None |
| | | I had mild swelling/bloating of the tummy |
| | | I had moderate swelling/bloating of the tummy and I was not able to do some of the things I normally do (household chores, shopping) |
| | | I had severe swelling/bloating of the tummy and I was not able to care for myself (e.g. getting out of bed, bathing, dressing) |
| | | I had very severe swelling/bloating of the tummy and I was not able to care for myself (e.g. getting out of bed, bathing, dressing) |
| **Nausea/vomiting** | Have you felt sick (nauseous/queasy)? | No |
| | | I felt a bit sick but I was able to eat and drink **the same amount** and type of foods as usual |
| | | I felt quite sick and I ate or drank **less than usual** or changed what I ate or drank |
| | | I felt very sick and was **not able** to eat or drink |
| | Have you had sickness (vomiting)? | No |
| | | I have vomited 1-2 times in one day |
| | | I have vomited 3 - 5 times in one day |
| | | I have vomited 6 or more times in one day |
| **Other Symptoms** | **Do you have any other symptoms that you wish to report?** Please list up to three other symptoms using the text boxes below: 1) | *Free text* |
| | **If answered:** | Mild |

| | | |
|---|---|---|
| | **During the past two weeks:** What was the severity of this symptom at its worst? | Moderate<br>Severe<br>Very severe |
| **Symptoms detail** | Please feel free to describe any of your symptoms in more detail below (*optional*): | *Free text* |
| **Blood test** | Along with completing this questionnaire, you should get your blood tested for the CA125 tumour marker at your GP practice or local hospital **every three months.** You will receive a reminder **around two weeks before** every review date to arrange this. | |
| | **When** did you last have your blood tested for the CA125 tumour marker? If you are not sure, please estimate a date (this will help your medical team locate your most up-to-date results). | *Date required* |
| | **Where** did you last get a blood test for the CA125 tumour marker (e.g. name of GP surgery, hospital, or any other place)? | *Free text* |
| **Appointment Request** | Do you need to speak to your Gynaecology Clinical Nurse Specialist (CNS) before your next scheduled review date? | No<br>Yes |
