# Supplementary material for: Electronic patient-reported monitoring of symptoms during follow-up of ovarian cancer patients: a feasibility study
Source: BMC Cancer. 2022 Jul 2;22:726. doi: 10.1186/s12885-022-09817-5 (PMC9250717; doi:10.1186/s12885-022-09817-5)
Supplement: Supplementary file 5 [file 12885_2022_9817_MOESM2_ESM.docx]

Additional file 2: The system/s architecture and access to the ePRO data by the CNS staff

During the development phase of the ePRIME project, work was undertaken to amalgamate a previously used system (called QStore) with a new tracker system. This resulted in a system called PROMPT (patient reported outcome measures pathway tracker), which was linked to the patient-facing web-based questionnaire builder system called QTool [23]. The figure illustrates how each system is linked.

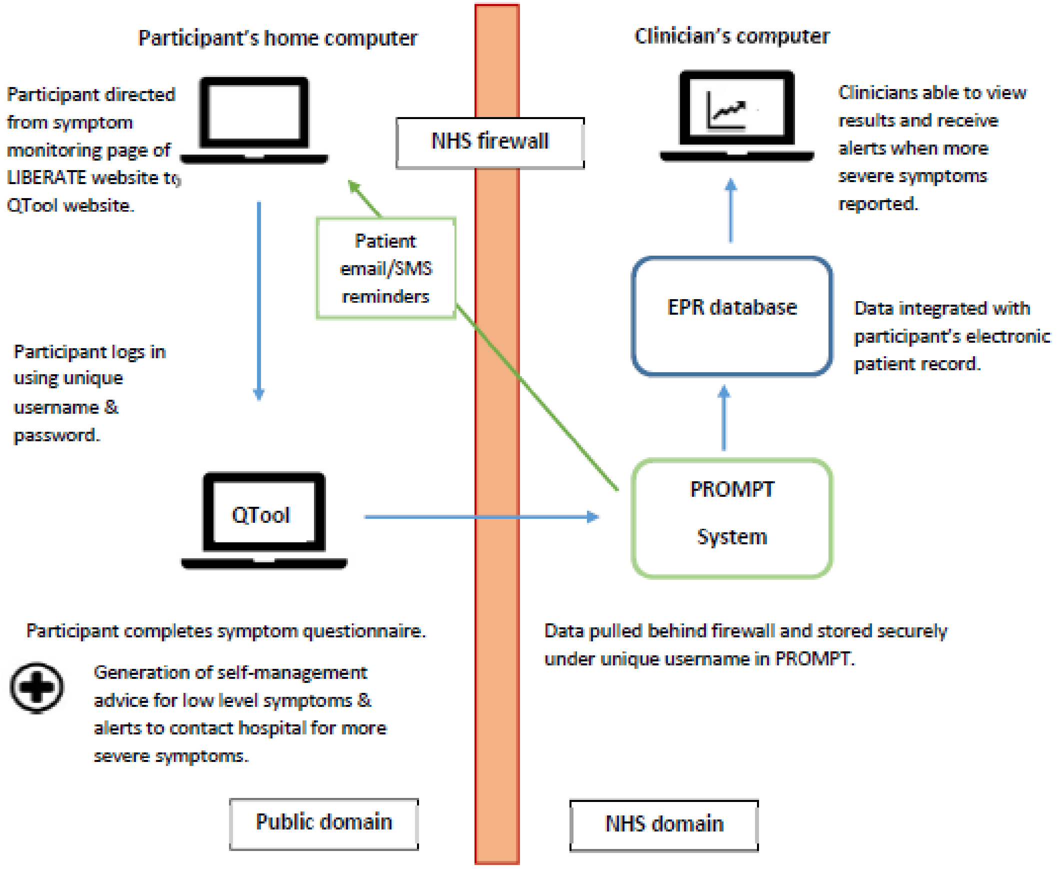

The new PROMPT system was designed to allow clinicians & researchers to monitor & manage patients who are completing their own PROs (Patient Reported Outcome) from home or outside of the clinic environment. Within PROMPT you can define individual patient pathways, and each pathway will consist of a scheduled set of investigations (or tasks) that the patient needs to complete (such as complete a QTool questionnaire, get a blood test etc.) at predefined time points within the pathway. The PROMPT system keeps track of these tasks, if and when they have taken place, and the system will also remind the patient (by text or email) to complete the investigation at the specified time points. The PROMPT system has a built-in alerts/notifications option which can be set to inform the clinician in real-time whenever a patient enters symptoms they wish to know about based on
