# Supplementary material for: Electronic patient-reported monitoring of symptoms during follow-up of ovarian cancer patients: a feasibility study
Source: BMC Cancer. 2022 Jul 2;22:726. doi: 10.1186/s12885-022-09817-5 (PMC9250717; doi:10.1186/s12885-022-09817-5)
Supplement: Supplementary file 6 [file 12885_2022_9817_MOESM3_ESM.docx]

Additional file 3: Computer use/experience of participants between the usual care and feasibility participants

	Audit (Before) N=45 †	Feasibility (After) N=24
**Computer usage: No. of years using a computer, N (%)**		
Never use	9 (20.0%)*	1 (4.2%)
Less than 1 year	0	0
1 – 2 years	0	0
2 – 5 years	2 (4.4%)	0
More than 5 years	28 (62.2%)	22 (91.7%)
Only with help	3 (6.7%)	1 (4.2%)
**Computer access: Main computer used, N (%)**		
Personal/Own	26 (57.8%)	18 (75.0%)
Family member	2 (4.4%)	1 (4.2%)
Friend	1 (2.2%)	0
Work	4 (8.9%)	3 (12.5%)
Other	0	2 (8.3%)
**Type of computer used, N (%)**		
Desktop	16 (35.6%)	11 (45.8%)
Laptop	15 (33.3%)	11 (45.8%)
Smart Phone	14 (31.1%)	9 (37.5%)
Tablet	18 (40.0%)	14 (58.3%)
Smart TV	5 (11.1%)	1 (4.2%)
Other	2 (4.4%)	2 (8.3%)
**Computer frequency of use, N (%) $**		
Daily	27 (60.0%)	19 (79.2%)
Weekly	3 (6.7%)	3 (12.5%)
Monthly	0	0
Very Rarely	3 (6.7%)	1 (4.2%)
**Computer activities, N (%)**		
Internet	31 (68.9%)	23 (95.8%)
Word and data processing	18 (40.0%)	13 (54.2%)
Games	10 (22.2%)	6 (25.0%)
Other	7 (15.6%)	2 (8.3%)
**Internet activities, N (%)**		
Don't use the internet	1 (2.2%)	1 (4.2%)
Email	28 (62.2%)	23 (95.8%)
Internet Banking	20 (44.4%)	19 (79.2%)
Online Shopping	21 (46.7%)	18 (75.0%)
Social Networking	19 (42.2%)	12 (50.0%)
News	20 (44.4%)	11 (45.8%)
Games	12 (26.7%)	7 (29.2%)
Streaming	10 (22.2%)	8 (33.3%)
Health Info	17 (37.8%)	15 (62.5%)
Other	3 (6.7%)	1 (4.2%)
**Computer confidence: How do you find using the computer in general? $**		
Easy	19 (42.2%)	16 (66.7%)
Sometimes difficult	12 (26.7%)	6 (25.0%)
Difficult	2 (4.4%)	1 (4.2%)
Impossible	0	0
**Computer enjoyment: Do you enjoy using a computer? $**		
Yes	21 (46.7%)	17 (70.8%)
No	3 (6.7%)	1 (4.2%)
Sometimes	9 (20.0%)	5 (20.8%)

* These participants did not answer the rest of the whole computer use questionnaire
† Three audit participants did not return the baseline demographic/computer use questionnaire
$ One pilot participant did not answer these items
