# Supplementary material for: Electronic patient-reported monitoring of symptoms during follow-up of ovarian cancer patients: a feasibility study
Source: BMC Cancer. 2022 Jul 2;22:726. doi: 10.1186/s12885-022-09817-5 (PMC9250717; doi:10.1186/s12885-022-09817-5)
Supplement: Supplementary file 7 [file 12885_2022_9817_MOESM4_ESM.docx]

Additional file 4: Percentage of scheduled versus unscheduled ePRO completions

	Overall n (%)	Hospital A	Hospital B
Number of scheduled / expected ePRO completions *	58 (74.4%)	11 (64.7%)	47 (77.0%)
Number of unscheduled/ additional ePRO completions #	20 (25.6%)	6 (35.3%)	14 (23.0%)

* 3 completions were completed after the patient had had their CNS review, but were still counted as scheduled/expected.

3 unscheduled completions were completed after the end of patient's time on the study (2 by one participant who had relapsed, 1 by another who had [illegible]
