# Supplementary material for: Electronic patient-reported monitoring of symptoms during follow-up of ovarian cancer patients: a feasibility study
Source: BMC Cancer. 2022 Jul 2;22:726. doi: 10.1186/s12885-022-09817-5 (PMC9250717; doi:10.1186/s12885-022-09817-5)
Supplement: Supplementary file 8 [file 12885_2022_9817_MOESM5_ESM.docx]

Additional file 5: Compliance with ePRO completions (proportion of completed/expected)

	Overall			Hospital A			Hospital B		
**Follow-up time point**	**Actual**	**Expected**	**Compliance %**	**Actual**	**Expected**	**Compliance %**	**Actual**	**Expected**	**Compliance %**
3 month	18	22	81.8	5	7	71.4	13	15	86.7
6 month	15	20	75.0	3	6	50.0	12	14	85.7
9 month	14	17	82.4	2	4	50.0	12	13	92.3
12 month	11	14	78.6	1	3	33.3	10	11	90.9
