# Supplementary material for: Electronic patient-reported monitoring of symptoms during follow-up of ovarian cancer patients: a feasibility study
Source: BMC Cancer. 2022 Jul 2;22:726. doi: 10.1186/s12885-022-09817-5 (PMC9250717; doi:10.1186/s12885-022-09817-5)

Additional file 6: Severity of symptoms reported amongst the feasibility study online responses

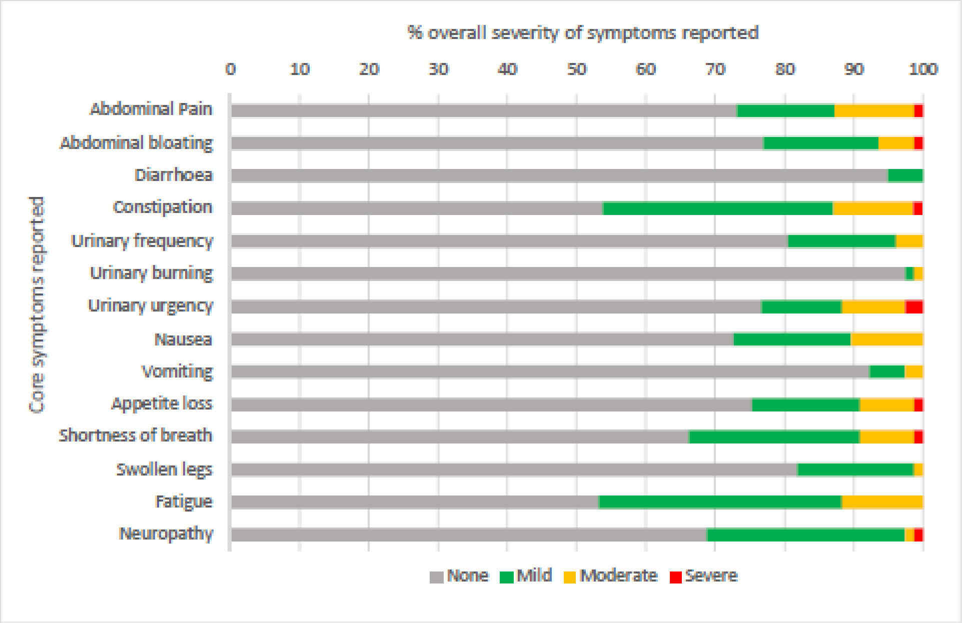

Supplement: Supplementary file 9 [file 12885_2022_9817_MOESM6_ESM.docx]
