# Supplementary material for: Electronic patient-reported monitoring of symptoms during follow-up of ovarian cancer patients: a feasibility study
Source: BMC Cancer. 2022 Jul 2;22:726. doi: 10.1186/s12885-022-09817-5 (PMC9250717; doi:10.1186/s12885-022-09817-5)
Supplement: Supplementary file 10 [file 12885_2022_9817_MOESM7_ESM.docx]

## Additional file 7: Overall collective severity levels across all completions

| | Total number of each symptom response across all symptoms (%) |
|---|---|
| None / Not experiencing symptom | 822 (75.3%) |
| Mild | 186 (17.0%) |
| Moderate | 66 (6.0%) |
| Severe | 8 (0.7%) |
| Very Severe | 0 |
| Missing | 10 (0.9%) |
