# Supplementary material for: Electronic patient-reported monitoring of symptoms during follow-up of ovarian cancer patients: a feasibility study
Source: BMC Cancer. 2022 Jul 2;22:726. doi: 10.1186/s12885-022-09817-5 (PMC9250717; doi:10.1186/s12885-022-09817-5)
Supplement: Supplementary file 11 [file 12885_2022_9817_MOESM8_ESM.docx]

Additional file 8: Case study example of a patient's use of system and presentation in electronic patient record for clinicians to review

Figure 8a and 8b (tabular and graphical screenshots below) relate to a case of a 60 year-old woman diagnosed in 2018 with stage 3C high-grade serous fallopian tube adenocarcinoma, with no gBRCA mutation detected. The patient was treated with primary surgery, followed by chemotherapy until December 2018. In mid-January 2019 she was recruited to ePRIME and was on study until relapse was detected and confirmed in December/January 2020.

Figures 8a and 8b present screenshots of the display in the Electronic Patient Record (EPR). This provides an example of how patient self-reports appear in the EPR. Figure 8a is a tabular format and shows how scores appear and that patients can add other free-text information or details about symptoms they are experiencing into the system. This additional information can then be viewed by staff by them hovering over the start of the text.

Apart from missing the 3 month ePRO report when the online system was unavailable in April 2019, the patient was fully compliant with the reminders to complete 3 monthly ePRO reports and obtain

blood tests at her general practice. At her 9 month review at the end of September 2019, she was asymptomatic (apart from longstanding mild shortness of breath and mild leg swelling for which she was seeing the lymphedema team), but her CA125 had risen from 14 to 34, and therefore it was decided to repeat the CA125 in 4 weeks. In November 2019 she completed an 'unscheduled' ePRO report 4-5 weeks after the 9 month completion, indicating new symptoms of mild 'occasional' abdominal pain, mild 'almost constant' bloating, and mild shortness of breath, which generated an alert to the clinical nurse specialist team. The CNS promptly rang her to discuss her symptoms, where she also said she was 'feeling full'. She was due to have her repeated CA125 taken, which was done that week and showed a further rise to 52. Patient was booked for CT scan which occurred in December and showed new measurable disease with peritoneal, nodal and suspected left groin metastatic deposit. Patient completed a further ePRO report in January 2020 a few days before her outpatient appointment with the consultant where the relapse was confirmed and communicated to the patient.

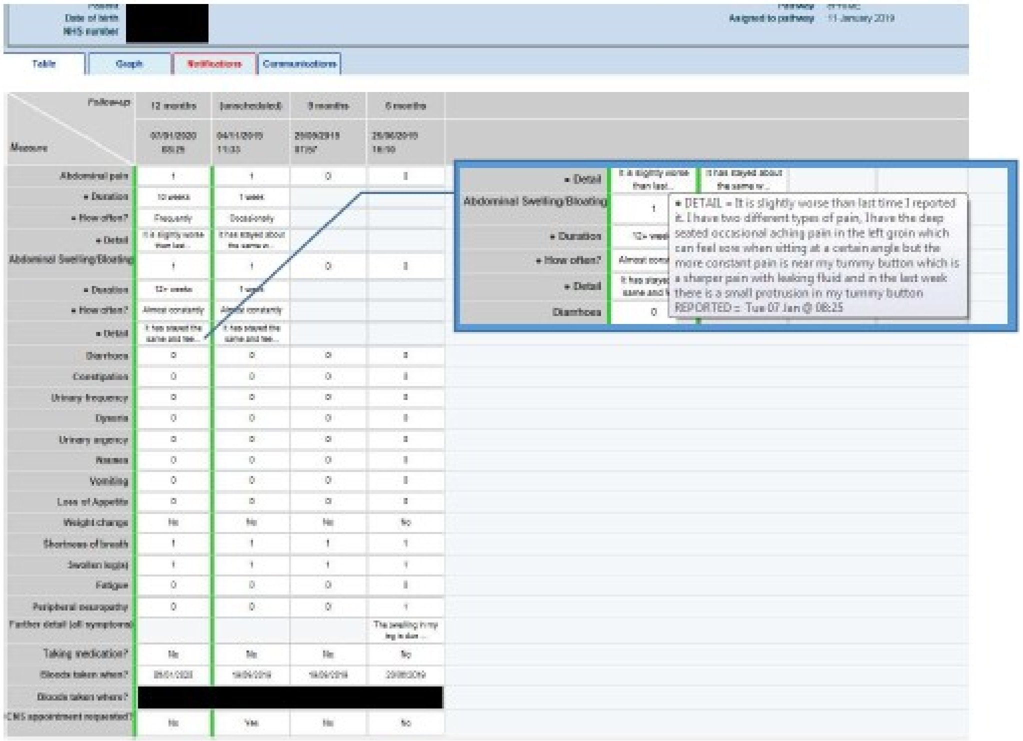

Additional file 8a – Tabular format, contains free text on additional symptoms or detail that patient wishes to add (most recent completion is on the left)

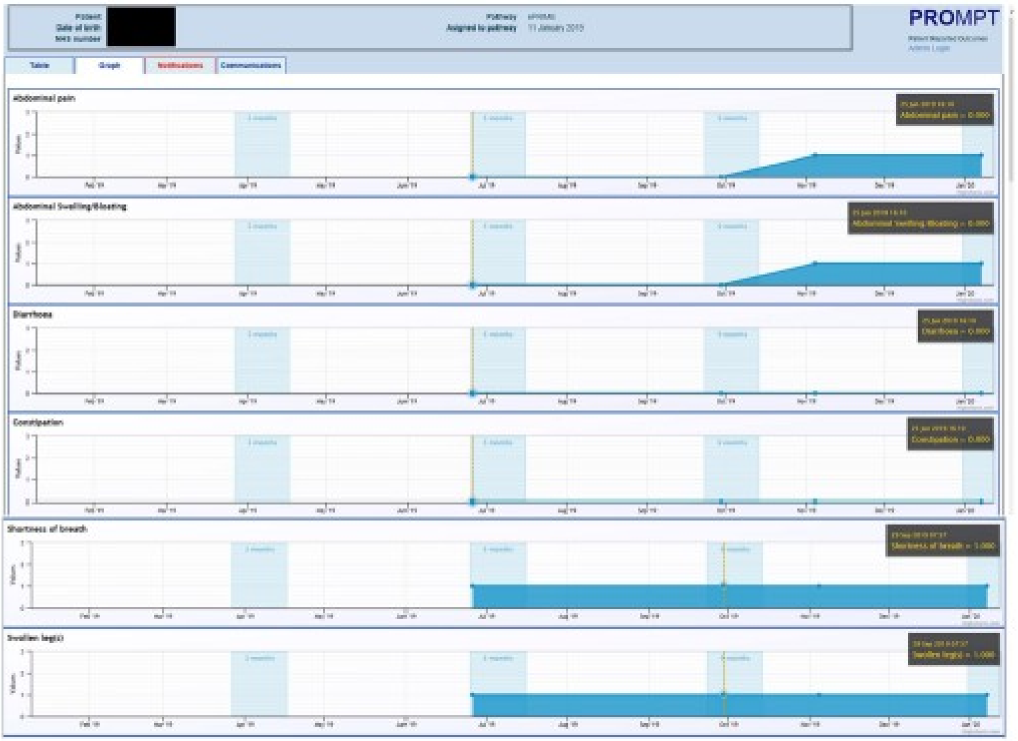

Additional file 8b – Graphic format to track symptoms over time (most recent completion is on the right)
