# Supplementary material for: Electronic patient-reported monitoring of symptoms during follow-up of ovarian cancer patients: a feasibility study
Source: BMC Cancer. 2022 Jul 2;22:726. doi: 10.1186/s12885-022-09817-5 (PMC9250717; doi:10.1186/s12885-022-09817-5)
Supplement: Supplementary file 12 [file 12885_2022_9817_MOESM9_ESM.docx]

Additional file 9: Overall reported holistic needs across all ePRO completions (76* full reports)

	Overall	Hospital A (out of 16 reports)	Hospital B (out of 60 reports)
Emotional (i.e. Worrying and anxiety)	30 (39.5%)	7 (43.8%)	23 (38.3%)
Confidence	6 (7.9%)	0	6 (10.0%)
Work/Employment	6 (7.9%)	1 (6.3%)	5 (8.3%)
Financial	6 (7.9%)	0	6 (10.0%)
Travel insurance	4 (5.3%)	2 (12.5%)	2 (3.3%)
Family	5 (6.6%)	0	5 (8.3%)
Psychological (e.g. depression)	10 (13.2%)	0	10 (16.7%)
Relationships	3 (3.9%)	0	3 (5.0%)
Sexual	6 (7.9%)	0	6 (10.0%)
Number of reports with holistic needs indicated	38 (50.0%)	9 (56.3%)	29 (48.3%)
Number of individual holistic needs	76	10	66

** 2 completions were partially completed, and the holistic needs section was not completed*
